# Increased tensile strength induced by the precipitation of nanocrystals for welding joints of Zr-based amorphous alloys

**DOI:** 10.1016/j.heliyon.2024.e35005

**Published:** 2024-07-22

**Authors:** Nannan Wang, Xiaohui Kang, Wumeng Liu, Wenjie Wu, Kexu Ren, Xiaohui Bao

**Affiliations:** a Civil Aviation Flight University of China, Guanghan, 618307, China; b Luoyang College, Civil Aviation Flight University of China, Luoyang, 471000, China; c Key Laboratory of Flight and Operation of General Aviation Training, Luoyang, 471000, China; d School of Materials Science and Engineering, Henan University of Science and Technology, Luoyang, 471000, China

**Keywords:** Amorphous materials, Welding, Laser processing

## Abstract

Zr-based amorphous alloys have attracted intensive attention for applications because of their excellent mechanical property. However, the welding process is inevitable for some special cases, such as the obtain of large size structure parts. It is significant to clarify the influence of introduced welding joints on mechanical properties in Zr-based amorphous alloys. Herein, the increased tensile strength of welding joints in Zr-based amorphous alloys is demonstrated by choosing a suitable initial temperature of Cu cooling fixtures for pulsed laser welding. It is found that an optimized tensile strength is observed when the initial temperature is −20 °C. With the decrease of the initial temperature from 10 to −30 °C, the tensile strength shows a trend of first increasing and then decreasing. Combined with the characterization of microstructures, it can be concluded that the increased tensile strength results from the precipitation of nanocrystals in the heat affected zone. Thus, our results provide a method to improve the mechanical property by controlling the microstructures of the heat affected zone in welding joints of Zr-based amorphous alloys.

## 1 Introduction

Amorphous alloy is a kind of alloy formed by superquenching solidification of alloy solution and has a long-range disordered and short-range ordered amorphous structure. It has no grains and grain boundaries and similar to glass in structure. Thus, it is also known as metal glass [1]. Ascribed to unique microstructures, amorphous alloys have better physical, chemical and mechanical properties than traditional crystalline alloys. It has presented broad application prospects in the fields of power transmission [1], 3C electronics [2], nuclear energy [3], medical devices [4], sporting goods and aerospace [5]. After decades of rapid development, a variety of systems of amorphous alloys have been prepared, There are mainly iron based amorphous alloy [6], Zr-based amorphous alloy [7], Ti-based amorphous alloy [8], Cu-based amorphous alloy [9], Mg-based amorphous alloy [10], Al-based amorphous alloy [11], Pb-based amorphous alloy [12], Co-based amorphous alloy [13], Ni-based amorphous alloy [14], etc. Zr-based amorphous alloys have been widely studied and applied because of their excellent amorphous forming ability and mechanical property.

The history of amorphous alloys can be traced back to the 1930s, which is first obtained by the evaporation deposition method [15]. The mercury supercooling experiment is proposed in 1952 [16], which provides the theoretical basis for amorphous alloys. The micron-scale Au–Si amorphous thin strips are prepared by using the chilling method in 1960 [17], which creates the history of melting quench cooling for the preparation of amorphous alloys. The millimeter-scale amorphous alloys of Pd–Cu–Si system are prepared by Cu die suction in 1973 [18], bringing amorphous alloys into the block era. Since then, scientists from various countries have tried different components to make larger amorphous alloys. For example, a massive amorphous alloy Pd_40_Ni_10_P_20_Cu_30_ is developed in 1989 [11], which has strong glass formation ability and a critical size of 75 mm. Since the 1990s, the research of bulk amorphous alloys has been developed rapidly and more systems of amorphous alloys have been achieved. The performance of the bulk amorphous alloys has become more and more excellent. In this case, some other materials are added into the amorphous alloy for maintaining the amorphous properties while increasing the toughness of the amorphous materials. For example, ceramic particles and metal particles are added into the amorphous alloy for the first time to obtain a particle-reinforced amorphous composite material [19], which has better room temperature toughness than traditional amorphous alloys. The tungsten fiber is added to amorphous alloy for the first time to prepare tungsten fiber-toughened Zr-based amorphous composite material [20], which has better plastic deformation ability than traditional amorphous material. The strength and plastic strain of Zr_53_Ti_5_Ni_10_Cu_20_Al_12_ amorphous alloy can be increased after the process of heat treatment [21], ascribed to the produced nanocrystals.

Meanwhile, the joining technology of Zr-based amorphous alloys are crucial for achieving a larger size, which presents potential applications in machinery manufacturing, aerospace and biomedicine [[22], [23], [24]]. Among the many existing welding methods [[25], [26], [27], [28], [29]], laser welding is a common method for amorphous alloys, which can ensure faster cooling and inhibit crystallization of welding joints effectively. The research of laser welding of amorphous alloys is mainly focused on the detection of crystal reduction and design of crystal composition. Wirginia et al. [30] obtained Fe-based amorphous alloy plates with specific compositions by die-casting method and carried out laser welding experiments. The hardness and mechanical properties of fusion zone (FZ) are similar to those of metallic glass. Crystallization occurs in the heat affected zone (HAZ) and the mechanical properties of welding specimens decrease to compare with base metal (BM). Wirginia et al. [31] prepared Zr-based amorphous alloy plates and carried out laser welding experiments. The experimental results are similar to those of Fe-based amorphous alloys and crystallization occurs in the HAZ. Wirginia et al. [32] tried to suppress crystallization in HAZ. They used a semiconductor cooling module, so there was no crystallization of HAZ during the welding process, resulting in a high-quality welding joint. Takuya et al. [33] used fiber lasers with high power density and increased heat dissipation to perform laser welding on Ni-based amorphous foils and obtained amorphous welding joints with excellent mechanical strength. Alavi et al. [34] used Fe-based amorphous alloy powder as filler material for laser welding of C1010 steel, and found that fine cells were formed in FZ. Thus, it is significant to clarify the influence of welding process on the mechanical property in Zr-based amorphous alloys, which can provide an insight for the improvement of mechanical property in welding joints.

In this work, we demonstrate the preparation of welding joints in Zr-based amorphous alloys by using pulsed laser welding. The relationship between initial temperature of Cu cooling fixtures and tensile strength of welding joints is revealed by adjusting the temperatures from 10 to −30 °C. The mechanical property is characterized and the optimized tensile strength is obtained by choosing a suitable initial temperature. Combined with characterizations of microstructures in HEA, it is found that moderate precipitation of nanocrystals in HAZ can be the main factor for the increased tensile strength.

## 2 Experimental methods

**Fig. 1:**
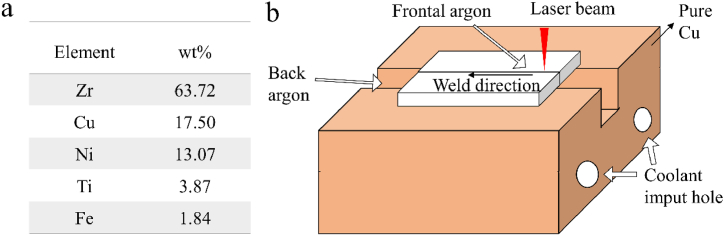
(a) Composition of Zr-based amorphous alloys. (b) Schematic diagram of laser welding.

The sheet to be weld is cut into cuboid with a size of 50.0 mm*10.0 mm*2.0 mm by wire cutting. After removing the raw edge and oxide layer, the welding sheet is analyzed by Niton XL2 handheld spectral analyzer and the composition is shown in Fig. 1a. The welding process diagram is shown in Fig. 1b. The laser device is IPG brand YLS-6000 fiber laser. Some preliminary experiments for obtaining a high quality welding joint. Thus, the welding parameters are roughly determined as power 4000 W, welding speed 0.4 m/s, defocusing amount 0 mm, pulse frequency 167 Hz, pulse duration 4 ms, laser spot diameter 0.3 mm. The protective gas uses 99.99 % concentration of high purity argon, and the front gas pumping speed is 25 L/min, and the back gas pumping speed is 15 L/min. The heat input is one of main factors for regulating the mechanical properties. Thus, by controlling the initial temperature, the cooling speed of welding parts can also be adjusted. We conducted a series of parameters of initial temperatures, which are 10, 0, −10, −20, and −30 °C, respectively. They are marked by L1, L2, L3, L4 and L5, respectively. Pure Cu is used on the surface of the fixture in contact with the material to be welded. The chosen of different initial temperatures is expected to affect microstructures of the welding joints, ascribed to the different cooling gradient. The microstructure is observed using 4XC metallography and Zeiss EVO 18 scanning electron microscope (SEM). Transmission electron microscopy (TEM) images are obtained by using FEI Tecnai G2 F20. Using the HV-1000Z microhardness tester tests Vickers hardness. The phase structure is analyzed by X-ray diffractometer (XRD, Cu rake, 40 kV). The tensile test is carried out by UTM4000 electronic universal testing machine, where the ASTM E8 is used.

## 3 Results and discussion

**Fig. 2:**
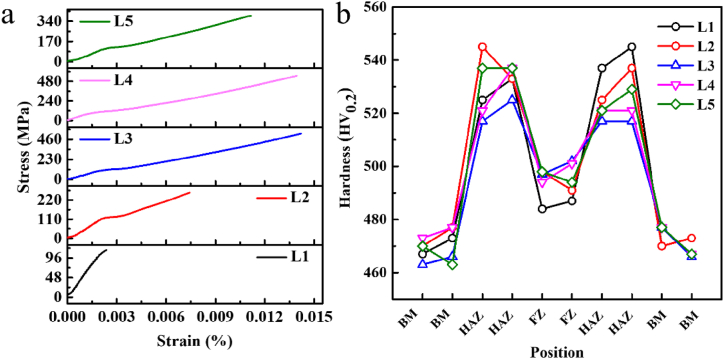
(a) Tensile curves for welding joint under different initial temperatures. (b) Microhardness of different parts of laser welding joint.

The tensile testing is shown in Fig. 2a, where the optimized tensile strength is observed for the sample welded at −20 °C and the value is 550 MPa. The tensile strength for samples welded at −10 °C and −30 °C are 527 and 386 MPa, respectively. The tensile strength for samples welded at 10 °C and 0 °C are 115 and 263 MPa, respectively. Such a result is similar with the research about the adjustment of laser power, which presents the trend of first increasing rapidly and then decreasing with the increasing of laser power [35]. The higher initial temperature decreases the heat transfer by decreasing the temperature gradient, inducing the larger heat input in welding region [36]. The lower initial temperature can not annihilate the defects in welding joints, which can also induce the decrease of tensile strength. The characterization of microhardness is shown in Fig. 2b. The testing direction was from the base material on one side to the heat affected zone, to the weld center to the heat affected zone and the base material on the other side. It can be observed that the region with the highest microhardness is in the HAZ with a microhardness of about 525 HV_0.2_, which could result from the appearance of nanocrystals in the HAZ. The region with the lowest microhardness is 470 HV_0.2_, which could result from non-crystalline based material (BM). The microhardness of the fusion zone (FZ) zone is between the HAZ and the BM, about 495 HV_0.2_. The trend is on the contrary to compare with the friction stir welding for the third-generation alloy (AA2198-T8), where BM presents the highest hardness [37]. The microhardness of the HAZ in different samples is relatively unstable due to the inhomogeneous mixing degree of nanocrystals and amorphous phase.

**Fig. 3:**
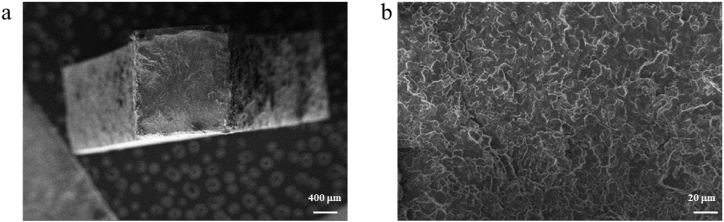
(a) Fracture morphology of tensile specimen for L4 and (b) corresponding enlarged view.

After the tensile test was completed at room temperature, the fracture morphology is observed in Fig. 3a and b. The tensile fracture of the welding specimen shows the normal fracture perpendicular to the tensile axis. The macroscopic morphology of the fracture shows that the fracture is relatively flat, which presents a typical river vein and no vein structure. It can be judged as a brittle cleavage fracture mode. The droplet material is not observed, which is different from the droplet shape with obvious amorphous fracture. Therefore, the fracture is HAZ with severe crystallization. The crack is located at the junction of the HAZ and BM. This is mainly because the boundary between the BM and HAZ is obvious and the temperature gradient in this region is obvious. The temperature of BM near the BM slowly drops, leading to crystallization and interfacial embrittlement. The cracks appear preferentially in this position. According to the crystallization characteristics of amorphous alloys, the mechanism of fracture in the HAZ can be explained. The internal microstructure of amorphous alloy is the disordered arrangement of atoms, and the internal microstructure of crystalline alloy is the ordered arrangement of atoms. The density of amorphous alloy with the same composition is lower than that of crystalline alloy, and there is a greater stress between the grain and the amorphous matrix in the heat affected zone. Thus, the tensile fracture is in this region.

**Fig. 4:**
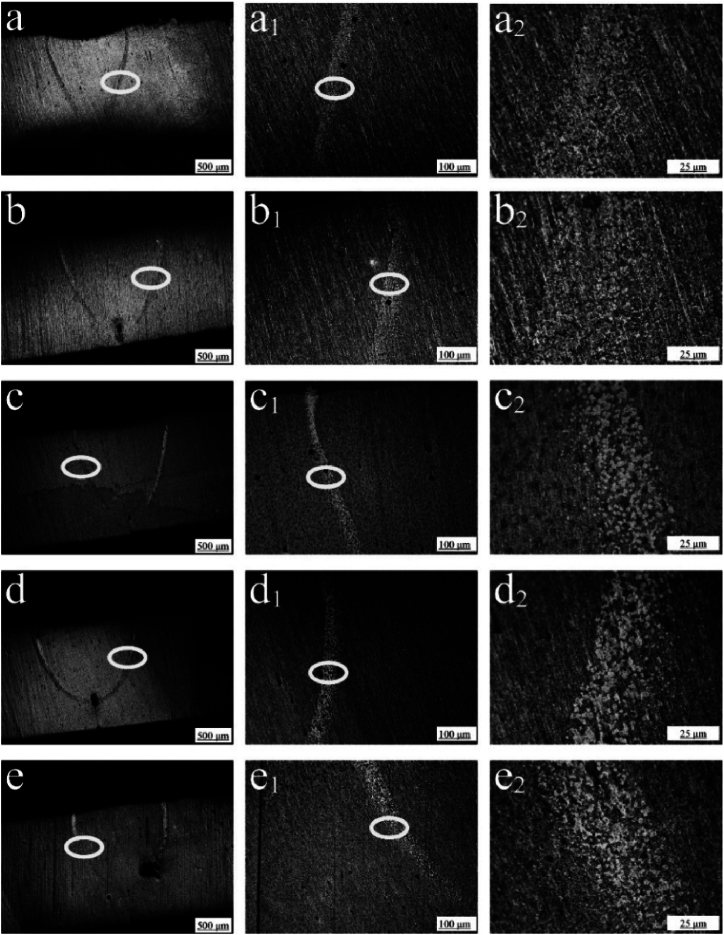
(a–e) Cross-section metallographic diagram of the welding sample for L1, L2, L3, L4 and L5, respectively. Local enlarged view of the heat affected zone of the welding sample under (a1-e1) 100 times and (a2-e2) 400 times for L1, L2, L3, L4 and L5, respectively.

To explore the mechanism of optimized tensile strength under the condition of −20 °C, Fig. 4 shows the optical microscope images of the welding joint under different magnifications. It can be observed that there is a narrow HAZ for all samples, and the upper middle part of the HAZ is wider than the lower part of the HAZ. With the decrease of the initial temperature from 10 to −20 °C (Fig. 4a–d), it can be found that the penetration depth is reduced in a small amplitude and the width of the lower part of the heat affected zone is significantly reduced. When the temperature decreases to −30 °C (Fig. 4e), the lower part of the heat affected zone of L5 has disappeared. Because the upper part of the heat affected zone of the welding joint is not in direct contact with the Cu fixture, the heat affected zone is wider than the lower part. Because the heat input is controlled as much as possible, there is relatively little crystallization. The coexistence of nanocrystals and amorphous crystals can be observed in parts of the heat affected zone. As the temperature of the Cu fixture decreases, it is observed that the grain size of the heat affected zone is smaller and more dispersed, with little overlap. The heat input during the welding process is a major factor affecting the width of the HAZ. The higher the heat input, the greater the HAZ width. An increase in the width of the HAZ usually leads to a decrease in the mechanical properties of the joint. The appearance of larger grain sizes can also result in less strength and toughness of the joint. By controlling the initial temperature, an optimized heat input is obtained, where the precipitation of nanocrystals in the heat affected zone can increase the tensile strength of welding joints. The crystallization can also be regulated by controlling the heat input induced by the laser power and welding velocity, where the optimized flexural strength can be obtained by choosing a suitable welding speed [36].

**Fig. 5:**
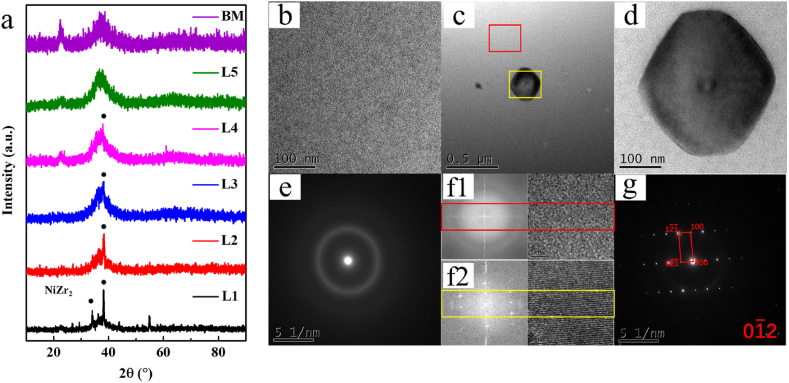
(a) XRD patterns of the base metal and each welding joint. (b–g) Transmission electron microscopy image of the HAZ of the welding joint.

Fig. 5a shows the results of XRD for all samples. A steamed bun peak is observed for the BM, indicating the disordered amorphous structure. Then, it is observed that the degree of crystallization gradually decreases with the decreased temperatures from 10 °C at −10 °C (for the samples L1 to L3). The degree of crystallization stabilizes at a low level after −10 °C (for the samples L4 and L5). The precipitation of NiZr_2_ phase can be observed from the results of XRD. Thus, The NiZr_2_ phase is observed in samples of L1-L4 and the steamed bun peaks appear gradually from the sample of L2 to L4, indicating the coexistence of amorphous alloys and nanocrystals. The classical steamed bun peaks are observed in L5 and BM. Fig. 5b–f shows the TEM images for the sample of L4. The disordered amorphous structure near the joint is observed in Fig. 5b and the nanocrystal (∼300 nm) is observed in Fig. 5c and d for the weld region. Accordingly, the diffraction rings are observed in Fig. 5e and f1. Obvious diffraction spots are found in Fig. 5f2 and the crystallized structure is labeled as the NiZr_2_ phase of the crystal band axis [−112] by combining with XRD (Fig. 5g). The main reason of the crystallization is the energy provided by the heat input in the welding process. By the nucleation and growth process, the nanocrystal can be observed in the HEZ of welding joints, which contributes the increased tensile strength.

## 4 Conclusions

In this work, we demonstrated the optimized tensile strength for welding joints of Zr-based amorphous alloys by controlling the temperature of the cooling fixture. A welding joint with excellent mechanical properties can be obtained at a cooling fixture temperature of −20 °C. It is found that the improvement of cooling conditions can significantly reduce the size of the heat affected zone of the welding joint. By combining with the characterization between microstructures and properties, it is concluded that the increased performance is ascribed to the appropriate precipitation of NiZr_2_ crystals in the HAZ. Crystallization mainly occurs in the heat affected zone. The average microhardness of the heat affected zone was the highest, about 530HV_0.2_, while the average microhardness of the base material was the lowest, about 475HV_0.2_. The average microhardness of the weld seam was about 498HV_0.2_. Ascribed to the advantages of high strength and corrosion resistance of Zr-based amorphous alloy, it has been widely used in aerospace, automotive industry and other fields. However, for obtaining a large size or complex structural part, the welding technology can be a convenient method. Thus, our results can provide an understanding for the improvement of mechanical property in welding joints and give a design reference for the process of pulsed laser welding.

## Data availability statement

Data will be made available on request.

## CRediT authorship contribution statement

**Nannan Wang:** Writing – original draft, Conceptualization. **Xiaohui Kang:** Visualization, Formal analysis. **Wumeng Liu:** Validation, Investigation. **Wenjie Wu:** Software, Methodology. **Kexu Ren:** Writing – review & editing, Visualization, Formal analysis. **Xiaohui Bao:** Writing – review & editing.

## Declaration of competing interest

The authors declare that they have no known competing financial interests or personal relationships that could have appeared to influence the work reported in this paper.
